# Accumulation of protein aggregates induces autolytic programmed cell death in hybrid tobacco cells expressing hybrid lethality

**DOI:** 10.1038/s41598-019-46619-5

**Published:** 2019-07-15

**Authors:** Naoya Ueno, Megumi Kashiwagi, Motoki Kanekatsu, Wataru Marubashi, Tetsuya Yamada

**Affiliations:** 1 United Graduate School of Agricultural Science, Tokyo University of Agriculture and Technology, Tokyo, Japan; 2 Faculty of Agricultural Science, Meiji University, Kanagawa, Japan

**Keywords:** Plant immunity, Plant molecular biology, Plant physiology

## Abstract

Hybrid cells of *Nicotiana suaveolens* x *N. tabacum* grow normally at 36 °C, but immediately express lethality due to probable autoimmune response when transferred from 36 to 28 °C. Our recent study showed that the temperature-sensitive lethality of these hybrid cells occurs through autolytic programmed cell death (PCD). However, what happens in hybrid cells following the induction of autoimmune response to autolytic PCD is unclear. We hypothesized that accumulation of protein aggregates in hybrid cells induces autolytic PCD and examined detergent-insoluble protein (protein aggregates) isolated from hybrid cells expressing lethality. The amount of insoluble proteins increased in hybrid cells. Sodium 4-phenylbutyrate, a chemical chaperone, inhibited both the accumulation of insoluble proteins and irreversible progression of cell death. In contrast, E-64, a cysteine protease inhibitor, accelerated both the accumulation of insoluble proteins and cell death. Moreover, proteome analysis revealed that proteasome-component proteins were accumulated specifically in cells treated with E-64, and proteasome activity of hybrid cells decreased after induction of lethality. These findings demonstrate that accumulation of protein aggregates, including proteasome subunits, eventually cause autolytic PCD in hybrid cells. This suggests a novel process inducing plant PCD by loss of protein homeostasis and provides clues to future approaches for elucidating the whole process.

## Introduction

Hybrid lethality is the phenomenon of death occurring in the hybrids of specific plant crosses^1^. The causative genes of hybrid lethality in plants have been identified and are commonly found to encode Resistance (R) proteins or R protein-interacting proteins^2–4^, suggesting that an autoimmune response via epistatic interaction of R genes is a common mechanism of hybrid lethality. Studies have shown that many defense-related genes are induced in *Nicotiana* hybrids and *Arabidopsis* hybrids exhibiting lethality^2,5^.

Hybrid seedlings and suspension cultured cells of *Nicotiana suaveolens* x *N. tabacum* are grown normally without any lethal symptoms when they cultured at 36 °C, but immediately express hybrid lethality when transferred from 36 to 28 °C, which is the optimal temperature for growth of the parents of the hybrids^6,7^. Physiological and cell biological features of programmed cell death (PCD) have been observed in these hybrid seedlings and cells expressing temperature-sensitive lethality^7–9^. Yamada *et al*.^7^ reported that the cell death process in the temperature-sensitive lethality reaches a point of no return between 2 and 3 h after the hybrid cells are transferred from 36 to 28 °C. In our recent study on this lethality, we revealed that the cell death is autolytic PCD, which is characterized by tonoplast rupture and the subsequent rapid clearance of the cytoplasm^10^. However, little is known about what happens in hybrid cells due to induction of autoimmunity triggered by the R gene to autolytic PCD.

In the cells of *N. suaveolens* x *N. tabacum* exhibiting hybrid lethality, autophagy-related features such as the increases of monodansylcadaverine-stained structures and *autophagy-related* gene transcripts have been observed at early periods of autolytic PCD^10^. Autophagy is one of the major pathways for degrading cellular components and is primarily responsible for the degradation of most long-lived or aggregated proteins and cellular organelles^11^. Several reports show that autophagy decreases protein aggregation in animal cells^12^. In plants, various proteins, such as cytochrome b5-RFP aggregates^13^, insoluble ubiquitinated protein aggregates^14^, and inactive proteasomes^15^, are degraded by autophagy. In addition, protein aggregates are often observed as electron-dense bodies by transmission electron microscopy (TEM) analysis^13,16,17^. In hybrid tobacco cells harboring autophagy-related features, electron-dense bodies have frequently been detected in vacuoles^10^.

Protein aggregates are observed following separation from lysate as the detergent-insoluble fraction using low-speed centrifugation^14,18^. Protein aggregation occurs from oligomeric complexes of non-native conformers that arise from unfolded proteins trapped with partial misfolded states, whose hydrophobic interaction makes them increasingly larger, more stable, and less soluble during severe stress conditions^19,20^. In animals and yeast, aggregates lack the function of the protein and heavy accumulation of protein aggregates causes the induction of cell death^21–23^. Accumulation of protein aggregates can be experimentally inhibited by sodium 4-phenylbutyrate (PBA), a well-described chemical chaperone in animal and plant cells^24,25^, and E-64, a cysteine protease inhibitor that blocks autophagic degradation in vacuoles^26^, causes the accumulation of the degradative protein aggregates^13^. However, little has been reported on the involvement of the accumulation of protein aggregates in cell death in plants. Moreover, it is unclear what impact differing amounts of protein aggregates have on cell death.

Based on these findings, we hypothesized that protein aggregates accumulate in *N. suaveolens* x *N. tabacum* hybrid cells and consequently cause autolytic PCD. In this study, we first investigated the amount of proteins in the detergent-insoluble fraction isolated from hybrid cells. Then, we examined the effects of exogenous treatment of PBA and E-64 on the accumulation of insoluble proteins and the progress of cell death in these hybrid cells. Moreover, to clarify which types of proteins are aggregated in hybrid cells, we conducted proteome analysis on insoluble proteins.

## Results

### Accumulation of insoluble proteins in hybrid cells expressing temperature-sensitive lethality

**Figure 1:**
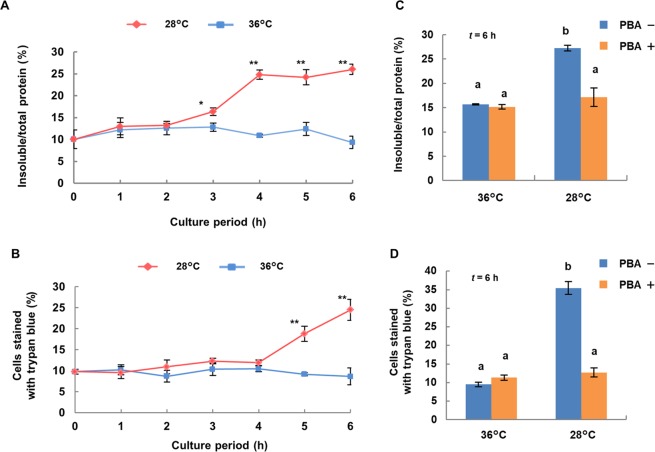
Temperature-dependent accumulation of insoluble proteins and expression of lethality in cultures of *N. suaveolens* x *N. tabacum* hybrid cells without and with PBA. Hybrid cells were cultured at 28 and 36 °C for 6 h. Time-dependent changes in (**A**) the percentage of insoluble protein to total protein extracted from hybrid cells and (**B**) the number of cells stained with trypan blue. For hybrid cells cultured at 28 and 36 °C for 6 h with PBA, (**C**) percentage of insoluble protein to total protein extracted from hybrid cells and (**D**) the percentage of insoluble protein and numbers of cells stained with trypan blue. Data shown are mean ± SE from replicate samples (*n* = 3) for each treatment. Asterisks indicate significant differences compared to level at 36 °C at the same time point (*P < 0.05, **P < 0.01, Student’s *t*-test). Different letters indicate significant differences as determined by the Tukey-Kramer test at *P* < 0.01.

Insoluble protein as a percentage of total protein in hybrid cells increased significantly in cells incubated at 28 °C starting at 3 h and then plateaued at 4 h. In contrast, cells incubated at 36 °C showed no change in insoluble protein level (Fig. 1A). The amount of total protein did not differ for cells incubated at 28 °C and at 36 °C (data not shown). To quantify the progression of cell death in hybrid cell cultures grown at 28 °C, the percentage of trypan blue-stained cells in individual cultures was determined. The percentage of trypan blue-stained cells was significantly higher in cultures incubated at 28 °C for 5 h than in cultures incubated at 36 °C (Fig. 1B).

To determine whether the percentage of insoluble proteins plays a role in the lethality in hybrid cells, we treated cultures with PBA, a chemical chaperone that prevents protein aggregation, and incubated the cultures for 6 h at 28 or 36 °C. After 6 h at 36 °C, there was no difference in cultures without or with PBA. Cultures at 28 °C showed that treatment with PBA significantly suppressed the accumulation of insoluble protein (Fig. 1C) and also the percentage of trypan blue-stained cells (Fig. 1D).

### Involvement of the accumulation of insoluble proteins in the determination of cell death in hybrid cells after induction of lethality

**Figure 2:**
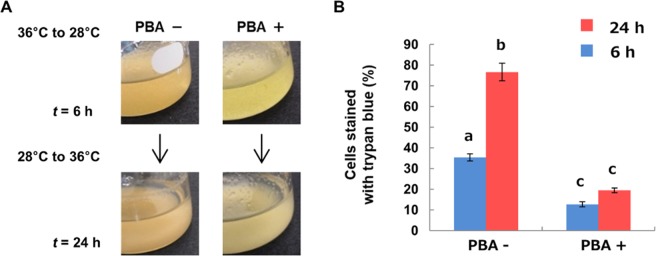
Effect of PBA on pathway of cell death of lethality in hybrid cells. (**A**) Hybrid cells cultured at 28 °C for 6 h with or without PBA were transferred to 36 °C for 24 h, and then (**B**) numbers of cells stained with trypan blue were measured. Data shown are the mean ± SE from different replicate samples (*n* = 3) for each treatment. The data at 28 °C for 6 h is from Fig. 1D. Different superscript letters indicate significant differences between cultures for each time period as determined by the Tukey-Kramer test at *P* < 0.05.

The point of no return is the point after which cell fate is irrevocably programmed cell death. In the case of *N. suaveolens* x *N. tabacum*, this occurs between 2 and 3 h of incubation at 28 °C following the transition from 36 °C. Specifically, hybrid cells transferred from 36 to 28 °C survived when they were returned to 36 °C up to 2 h after the transfer but died when they were returned to 36 °C at 3 h or later. In this experiment, in order to determine whether the accumulation of insoluble proteins contributes to the determination of cell death in hybrid cells, cells transferred from 36 to 28 °C were treated with PBA and returned to 36 °C at 6 h after the transfer. Up to 24 h after the hybrid cells were returned to 36 °C, cells treated with PBA remained green (Fig. 2A) and the percentage of trypan blue-stained cells treated with PBA was significantly lower than for untreated cells (Fig. 2B). In addition, the increase of trypan blue-stained cells was observed only in the without PBA treatment.

### Effects of E-64 on accumulation of insoluble proteins and temperature-sensitive lethality in hybrid cells

**Figure 3:**
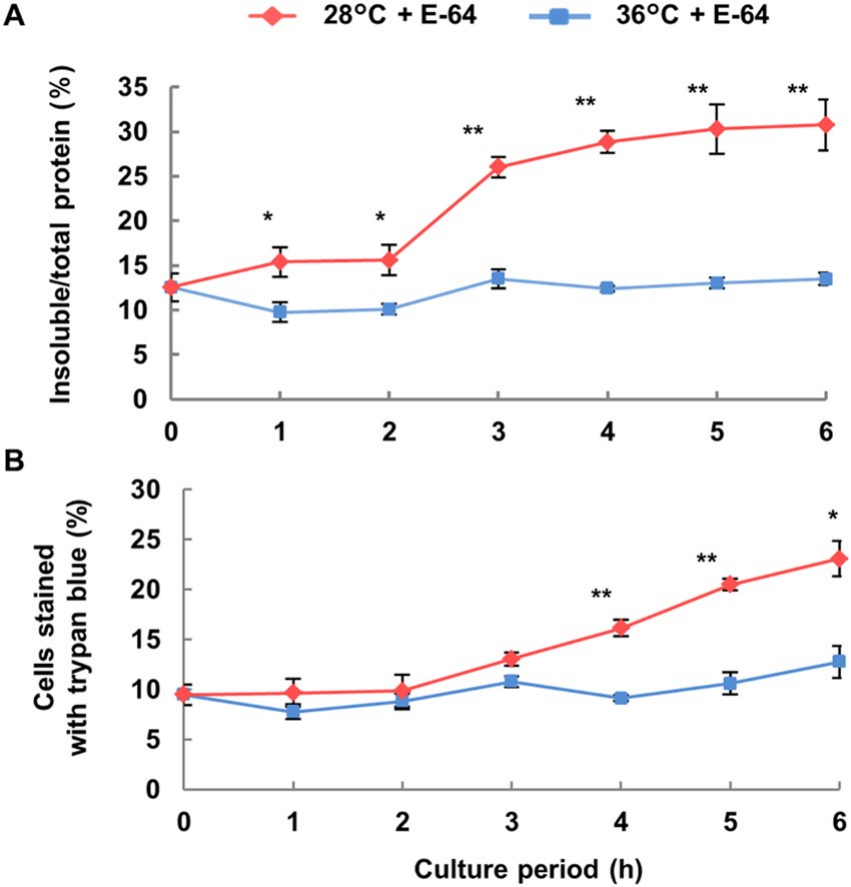
Effect of E-64 on temperature-dependent accumulation of insoluble protein and expression of lethality in hybrid cells. Hybrid cells were cultured with E-64 at 28 or 36 °C for up to 6 h. (**A**) The percentage of insoluble protein to total protein extracted from hybrid cells and (**B**) numbers of cells stained with trypan blue were measured. Data shown are the mean ± SE from different replicate samples (*n* = 3) for each treatment. Asterisks indicate significant differences compared with data at 36 °C (*P < 0.05, **P < 0.01, Student’s *t*-test).

We investigated the effects of E-64, which is an inhibitor of autophagic degradation, on insoluble proteins and the percentage of trypan blue-stained cells. Insoluble protein as a percentage of total protein in E-64-treated cultures increased significantly at 1 h incubation at 28 °C and then plateaued at 3 h. In contrast, for incubation at 36 °C, insoluble protein content did not markedly change (Fig. 3A). The percentage of trypan blue-stained cells was significantly higher in E-64-treated cultures at 28 °C from 4 to 6 h than in cultures incubated at 36 °C (Fig. 3B). Moreover, the time to detect a significant increase in insoluble protein and cells stained with trypan blue due to incubation at 28 °C was faster in E-64-treated cultures than in the untreated cultures (Figs 1A,B, 3A,B).

### Effects of E-64 on formation of electron-dense bodies in vacuoles of hybrid cells

**Figure 4:**
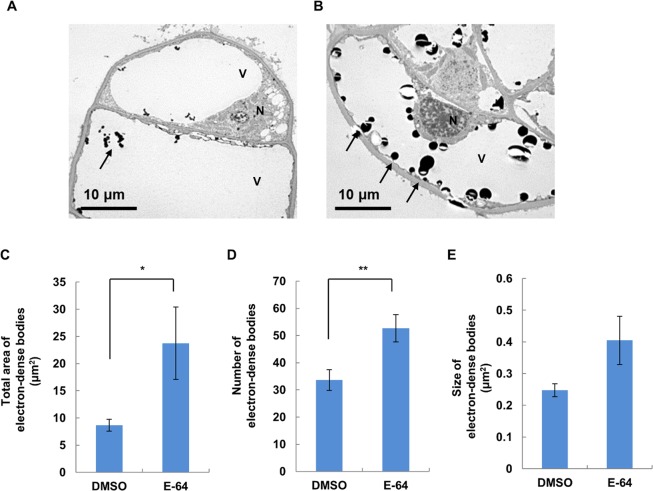
Effect of E-64 on accumulation of electron-dense bodies in vacuoles from hybrid cells incubated at 28 °C. Electron-dense bodies were observed in hybrid cells incubated at 28 °C for 3 h with (**A**) DMSO or (**B**) E-64. (**C**) Total area, (**B**) number and (**C**) size of electron-dense bodies in each TEM image calculated using Image J software. Data shown are the mean ± SE of more than 10 images from each treatment. Asterisks indicate significant differences from DMSO control (**P < 0.01, Welch’s *t*-test).

Insoluble protein increased significantly by 3 h with incubation at 28 °C in E-64-treated cultures while only a slight increase was observed at the same time point in untreated cultures (Figs 1A, 3A). Observation of the ultrastructure of hybrid cells cultured at 28 °C for 3 h with and without E-64 via TEM showed that electron-dense bodies were mainly detected in the vacuoles of hybrid cells in both cultures (Fig. 4A,B). For more accurate analysis, we measured the area, number and size of electron-dense bodies on TEM image analysis. The area and number of electron-dense bodies in each cell vacuole were significantly higher in E-64-treated cultures than in untreated cultures (Fig. 4C,D). The size of electron-dense bodies was also larger in E-64-treated cultures but not significantly greater (Fig. 4E).

### Proteome analysis of insoluble proteins

**Figure 5:**
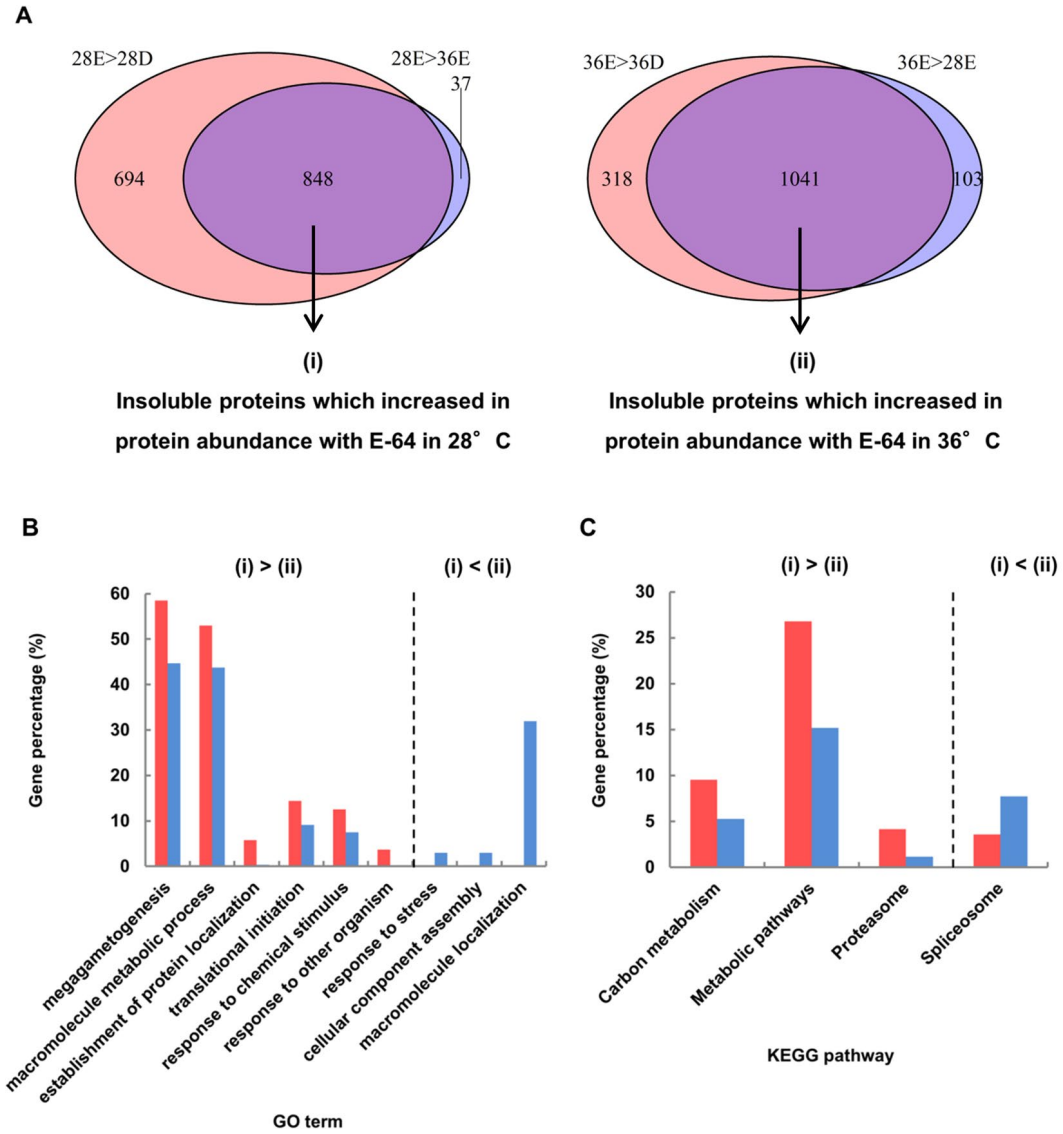
GO and pathway enrichment analysis of insoluble proteins in hybrid cells. (**A**) Venn diagram showing the number of identified insoluble proteins which increased in protein abundance with E-64 at 28 °C (i) and 36 °C (ii). The percentage of genes in each (**B**) GO and (**C**) pathway that was significantly overrepresented between (i) and (ii) (*q* < 0.05, Benjamini–Hochberg-corrected Fisher’s exact test) is shown.

To confirm what kind of insoluble proteins were generated and degraded in hybrid cells, especially with incubation at 28 °C in the presence of E-64 for 3 h, we carried out LC-MS/MS analyses of insoluble proteins extracted from four groups of hybrid cells incubated for 3 h with E-64 or DMSO: group 28D was incubated at 28 °C with DMSO; group 28E was incubated at 28 °C with E-64; group 36D was incubated at 36 °C with DMSO; group 36E was incubated at 36 °C with E-64. A total of 2462 proteins were identified by the analysis of four groups (Supplementary Table S1). Of these proteins, 1347 proteins were detected in 28D, 1673 proteins in 28E, 1247 proteins in 36D and 1500 proteins in 36E. Then, we identified insoluble proteins (i) insoluble proteins which increased in protein abundance with E-64 at 28 °C compared to without E-64 at 28 °C and with E-64 at 36 °C and (ii) insoluble proteins which increased in protein abundance with E-64 at 36 °C compared to without E-64 at 36 °C and with E-64 at 28 °C using the following criteria of %emPAI value: (i) 28E > 28D and 28E > 36E and (ii) 36E > 36D and 36E > 28E. As a result, 848 and 1041 proteins were identified in (i) and (ii), respectively, and were subjected to further analysis (Fig. 5A and Supplementary Tables S2 and S3).

**Figure 6:**
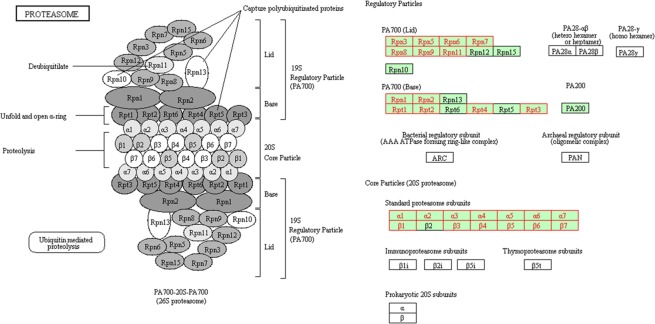
Pathway map of proteasome detected specifically in insoluble proteins at 28 °C with E-64 is shown in red. BLASTP best hits for *A. thaliana* proteins in *N. tabacum* proteins belonging to the proteasome pathway are presented in green.

GO analysis and pathways of the identified proteins were analyzed and compared between (i) and (ii) (see Supplementary Tables S4 and S5). GOs associated with translational initiation (GO:0006413), megagametogenesis (GO:0009561), response to chemical stimulus (GO:0042221), macromolecule metabolic process (GO:0043170), establishment of protein localization (GO:0045184) and response to other organisms (GO:0051707) were significantly overrepresented among the insoluble proteins which increased in protein abundance with E-64 at 28 °C (i) (Fig. 5B). From pathway analysis, carbon metabolism, metabolic pathways and proteasome were significantly overrepresented among the proteins (i) (Fig. 5C). Most of the proteasome component proteins (26 out of 34) were identified in (i) (Fig. 6). In contrast, GOs associated with response to stress (GO:0006950), cellular component assembly (GO:0022607) and macromolecule localization (GO:0033036) were significantly overrepresented among the insoluble proteins which increased in protein abundance with E-64 at 36 °C (ii) (Fig. 5B). In addition, only the spliceosome pathway was significantly overrepresented among the insoluble proteins (ii) (Fig. 5C).

### Proteasome activity in hybrid cells

**Figure 7:**
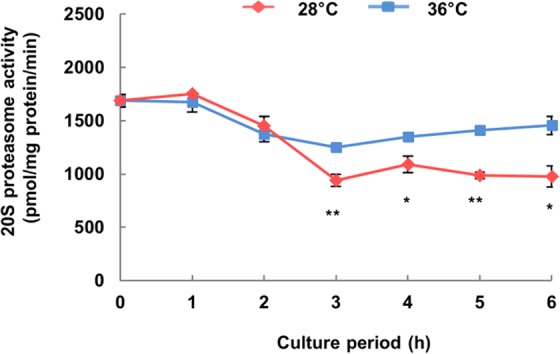
Time course of proteasome activity in hybrid cells expressing lethality. Proteasome activity was determined using Suc-LLVY-AMC as the substrate. Data shown are the mean ± SE from replicate samples (*n* = 3) for each treatment. Asterisks indicate significant differences compared to 36 °C (*P < 0.05, **P < 0.01, Student’s *t*-test).

From the proteome analysis of insoluble proteins, more proteasome component proteins were significantly detected among the insoluble proteins which increased in protein abundance with E-64 at 28 °C than at 36 °C. Then, we measured proteasome activity in hybrid cells incubated at 28 °C. Proteasome activity of hybrid cells was more significantly decreased in cultures exposed to 28 °C for 3 h than in cultures exposed to 36 °C (Fig. 7). In addition, proteasome activity was significantly reduced by MG-132, a proteasome inhibitor (Supplementary Fig. S1), so the exact detection of proteasome activity by the method used in this experiment was confirmed.

## Discussion

Insoluble protein as a percentage of total proteins in hybrid cells increased in incubation at 28 °C for 3 h before the progression of cell death as indicated by the increase of trypan-blue stained cells (Fig. 1A,B). PBA, a chemical chaperone, suppressed the increase of insoluble proteins at 28 °C for 6 h (Fig. 1C), but at the same time, no increase in trypan-blue stained cells was observed with PBA (Fig. 1D). Several reports demonstrate that PBA acts as a chemical chaperone that reduces protein aggregates and suppresses endoplasmic reticulum (ER) stress^24,25^. The point of no return during PCD is defined as the step beyond which the cell is irreversibly committed to die^27^, and PBA could allow the hybrid cells to survive, even after the point of no return (Fig. 2B,C). These results indicate that the increase in insoluble protein causes the irreversible cell death (autolytic PCD) in hybrid cells during hybrid lethality. Moreover, the increase in insoluble protein was considered to be attributable to the accumulation of protein aggregates in hybrid cells.

We used E-64, a cysteine protease inhibitor that blocks autophagic degradation in vacuoles^26^, to inhibit protein degradation in hybrid cells. Inoue *et al*.^26^ also showed that E-64 blocks autophagosome degradation in *Arabidopsis* and barley, which is followed by the accumulation of cytoplasmic inclusions within the central vacuole. Practically, the increase of insoluble proteins occurred 2 h earlier with E-64 than without (Figs 1A, 3A). For incubation at 28 °C for 3 h, insoluble protein was highly increased in E-64-treated cultures, and then, enlargement and increases in electron-dense bodies were detected in vacuoles of hybrid cells via TEM (Figs 3A, 4A). These results indicate that as protein aggregates increased, insoluble proteins are accumulated but also degraded by cysteine proteases in vacuoles. Thus, the increase in insoluble proteins is only apparent at 3 h without E-64 treatment. However, the increase of insoluble proteins at 3 h after induction of lethality even without E-64 indicates that accumulation of insoluble proteins exceeded the amount of endogenous degradation for insoluble proteins in hybrid cells. In addition, the increase of dead cells was observed 1 h earlier with E-64 than without E-64 (Figs 1B, 3B). This agrees with the finding that an increase in insoluble proteins induces autolytic PCD in hybrid cells during hybrid lethality.

From proteome analysis, we identified what kind of insoluble proteins showed increased in abundance in hybrid cells, specifically in the presence of E-64 at 28 °C, that were generated but subsequently degraded at the point of no return. Almost all proteasome-component proteins were identified as insoluble proteins from hybrid cells expressing lethality at 28 °C (Figs 5B, 6). Proteasome activity actually decreased significantly in hybrid lethality at 28 °C for 3 h (Fig. 7) at the same time that insoluble proteins were accumulated (Fig. 1A). The ubiquitin proteasome system (UPS) plays the critical role of recognizing and selectively degrading misfolded and damaged proteins to prevent potentially toxic effects of protein aggregation^28,29^. Therefore, these proteasome-component proteins were mostly aggregated and detected as insoluble proteins and because they caused impairment of UPS, insoluble proteins consequently increased.

In plants, a putative role for the proteasome in PCD has been suggested. Kim *et al*.^30^ showed that dysfunction of UPS by gene silencing of proteasome subunits activates PCD in plants. However, the reason why impairment of proteasome activity leads to cell death is poorly understood. One possibility is that protein aggregates accumulate by the dysfunction of UPS, leading to cell death as in our study. Generally, the accumulation of protein aggregates in ER lumen activates the ER-stress response^31^, and when it is prolonged, cell death occurs in plants^32^. Thus, it is possible that accumulation of protein aggregates by the impairment of proteasome activity causes persistent ER-stress and eventually leads to autolytic PCD in hybrid cells.

On the other hand, what are the reasons for proteasome activity impairing hybrid cells during hybrid lethality? It is known that protein aggregates can clog up the proteasome and inhibit proteolysis^21^, but why were protein aggregates first generated in hybrid cells during hybrid lethality? Protein aggregates in general comprise proteins that have been damaged by excessive production of reactive oxygen species (ROS)^33^. Mino *et al*.^34^ reported that some kinds of ROS are produced in large quantities in tobacco hybrids (*N. gossei* × *N. tabacum*) exhibiting lethality and they are involved in the lethality. From these studies, we considered the possibility that ROS generated after the induction of lethality in hybrid cells causes the formation of protein aggregates, which clog the proteasomes to the point that activity is impaired, which in turn leads to excess accumulation of protein aggregates, causing continuous ER-stress, and eventually leads to autolytic PCD in hybrid cells. Additional experiments are needed to further elucidate this hypothesis.

Proteome analysis of insoluble proteins also suggested that more proteins related to carbon metabolism and metabolic pathways aggregated and lost functionality during hybrid lethality at 28 °C than at 36 °C (Fig. 6B). After induction of lethality, hybrid cells underwent growth arrest and death. Therefore, the high abundance of proteins, which are responsible for normal growth such as metabolic reactions, might be damaged and aggregated, becoming insoluble. Spliceosomes, which are composed of small nuclear ribonucleoproteins that are required for proper RNA splicing, are more likely generated under normal growth conditions than stress conditions and are mainly aggregated and degraded. In our study, the spliceosome pathway was enriched with insoluble proteins in hybrid cells at 36 °C (Fig. 6B), but we have no mechanistic explanation for why spliceosomal proteins were specifically aggregated in healthy cells.

In conclusion, accumulation of insoluble proteins caused induction of autolytic PCD in hybrid cells. Moreover, we considered insoluble proteins as protein aggregates, and thus proposed that the aggregates, including proteasome subunits, eventually cause autolytic PCD in hybrid cells. This is the first evidence for the relationships between protein aggregates and autolytic PCD in plants.

## Materials and Methods

### Cell culture

The suspension cell line was established from cotyledonary segments of hybrid seedlings (*N. suaveolens* x *N. tabacum*) as described in Yamada *et al*.^35^. Hybrid cells were cultured at 36 °C and subcultures were made every 10 days. In all experiments, 3 mL of packed cell volume from cultured cells was transferred to incubation chambers set to 28 °C or 36 °C, as described in Masuda *et al*.^9^, and incubated for 6 h. At 1 h intervals, protein extraction and staining with 0.4% (w/v) trypan blue in phosphate-buffered saline (PBS) were conducted, and cell death was scored under a light microscope. The extent of cell death in hybrid cells was estimated based on the percentage of dead cells among all cells. To evaluate the effects of E-64 and PBA on protein aggregates and cell death of hybrid cells, the hybrid cell cultures were incubated at 28 or 36 °C in the same way in medium containing 10 µM E-64 (Peptide Institute) and 5 mM PBA (Cayman Chemical). As a control, we added the same amount of solvent for E-64 and PBA, dimethyl sulfoxide (DMSO) and distilled water respectively. To inhibit proteasome activity, the hybrid cell cultures were incubated in medium containing 10 µM MG-132 (Peptide Institute) dissolved in DMSO. The concentrations of all treatments were determined in optimization experiments prior to the experiments.

### Extraction of total protein and isolation of protein aggregates

Frozen cell samples (100 mg) were disrupted and homogenized with stainless steel beads and a TissueLyser LT (Qiagen) in 1 mL of protein extraction buffer (100 mM Tris-HCl, pH 8.0, 10 mM NaCl, 1 mM DTT, 1% Triton X-100, 0.2% β-mercaptoethanol) under ice-cold conditions. The total protein content in homogenates was determined on an aliquot of the homogenate using the RCDC Protein Assay (Bio-Rad Laboratories). Protein aggregates were isolated from the rest of the homogenates as the insoluble protein fraction using low-speed centrifugation as described in Zhou *et al*.^14^. Briefly, the homogenates were centrifuged at 2,200 × g for 5 min, the supernatant was discarded, and precipitates were resuspended twice more in 1 mL of the same extraction buffer followed by centrifugation to wash out the soluble proteins. The last pellets were resuspended with the same extraction buffer and used to calculate the amount of insoluble proteins using the RCDC Protein Assay. Using the amount of total proteins and insoluble proteins, the percentage of insoluble proteins was calculated.

### TEM

TEM imaging of cells was performed by Tokai Electron Microscopy Inc., as described in detail in Ueno *et al*.^10^. TEM images were randomly selected, and more than 10 images were analyzed to measure the total area, number, and size of electron-dense bodies in each image using ImageJ^36^.

### LC-MS/MS data acquisition of insoluble proteins

Insoluble proteins were isolated from hybrid cell cultures incubated at 28 or 36 °C for 3 h in medium containing E-64 or DMSO. Insoluble proteins (5 μg) were separated by SDS-PAGE on 15% polyacrylamide gels at 30 mA and stained by Coomassie brilliant blue R-250. The stained gels were divided equally into ten slices, dried in a vacuum centrifuge, and reduced with 10 mM DTT in 100 mM NH_4_HCO_3_ for 1 h at 56 °C, and then alkylated with 55 mM iodoacetamide in 100 mM NH_4_HCO_3_ at ambient temperature in the dark with occasional vortexing for 45 min. In-gel proteins were digested by 12.5 ng/μl Trypsin Gold (Promega) in 50 mM NH_4_HCO_3_ at 37 °C overnight. The peptides were extracted three times with 50% acetonitrile and 5% formic acid, and dried down by vacuum centrifuge.

LC-MS/MS analysis was conducted with an LTQ Orbitrap XL (Thermo Fisher Scientific) according to Yoshikawa *et al*.^37^. Peptide separation was carried out at room temperature on a C18 column at a flow rate of 100 nL/min with a 0%–80% gradient of acetonitrile. Mass spectra were recorded under data-dependent mode to automatically switch between Orbitrap-MS and linear ion trap-MS/MS acquisition with a centroid mode. Survey full-scan MS spectra (450 to 1500 m/z) were acquired in the Orbitrap with resolution set to 15,000 after accumulation to a target value of 500,000 in the linear ion trap. The top 10 precursor ions from each MS scan were isolated for fragmentation for every 0.2 s in the linear ion trap using collision induced dissociation at a target value of 30,000.

### LC-MS/MS data analysis

Data analysis was performed as described in Yoshikawa *et al*.^37^ with little modification. The raw data acquired by Xcalibur version 2.0.7 (Thermo Fisher Scientific) was converted to an mgf file by Proteome Discoverer version 1.1 (Thermo Fisher Scientific). A search was performed by MASCOT version 2.2.07 against the *N. tabacum* cv. TN90 protein database in NCBI RefSeq with the parameters of fixed modification and carbamidemethyl (Cys); variable modifications of oxidation (Met) and pyroglutamine; maximum missed cleavages of 2; peptide mass tolerance of 25 ppm; and MS/MS tolerance of 0.8 Da. The candidate peptides were identified as having significant homology (p < 0.05) and are referred to as “hits”. Further, we a set more strict criteria for protein assignment: any peptide candidate with an MS/MS signal number of <2 was eliminated from the “hits” candidates. For estimation of the absolute protein amount assigned by the database search, we used the exponentially modified protein abundance index (emPAI)^38^. The %emPAI (normalized amount of proteins) was used for further analysis.

### Gene ontology (GO) analysis

Blastp program was used to compare protein sequences of *N. tabacum* cv. TN90 from NCBI RefSeq and *Arabidopsis thaliana* from the TAIR10 database (E-value < 1e^−5^) to convert the protein ID of *N. tabacum* to blastp top-hit *A. thaliana* AGI code. The converted gene list was analyzed on the DAVID database (https://david.ncifcrf.gov/)^39^ to identify gene ontology (GO) classifications and the Kyoto Encyclopedia of Genes and Genomes (KEGG) pathways^40^. Using these data, Fisher’s exact test was conducted on R^41^ and the adjusted p-value by the Benjamini–Hochberg procedure (q-value) was calculated to determine which GO and pathway were significantly enriched in the different groups.

### Proteasome activity assay

Frozen cell samples (100 mg) were disrupted and homogenized with stainless steel beads and a TissueLyser LT in proteasome extraction buffer (50 mM Tris, pH 7.5, 150 mM NaCl, 2 mM DTT, 5 mM EDTA) under ice-cold conditions. After incubation on ice for 30 min, cell debris was removed by centrifugation at 20,000 × g for 15 min at 4 °C. The supernatant was immediately used for enzyme assays performed in reaction buffer (25 mM HEPES, pH 7.5, 2 mM DTT, 5 mM EDTA) incubated for 60 min at 37 °C. 50 μM Succinyl-L-leucyl-L-leucyl-L-valyl-L-tyrosine 4-methylcoumaryl-7-amide (Suc-LLVY-AMC) (Peptide Institute) was used as the substrate of proteasome; the amount of 7-amino-4-methyl-coumarin released was determined with a SpectraMax Paradigm Multimode Microplate Reader (Molecular Devices) based on fluorescence emission at 460 nm upon excitation at 360 nm. Protein content was determined using a Qubit Protein Assay Kit (Life Technologies) with a bovine serum albumin solution as the standard.

### Statistics

All the numeric data are reported as mean values with standard error (SE). Statistical analysis was performed with the Student’s *t*-test or Welch’s *t*-test for the comparison of two groups and the Tukey-Kramer test for multiple comparisons. Student’s *t*-test was carried out under the assumption of equal variance and Welch’s *t*-test was conducted for the assumption of unequal variance using the two-sample F-test. In comparisons of percentage data, statistical analysis was done following arcsine transformation. In all cases, differences with *P* < 0.05 were considered statistically significant.

## Supplementary information

Supporting Information

Dataset 1

Dataset 2

Dataset 3

Dataset 4

Dataset 5

## Data Availability

All data generated or analyzed during this study are included in this published article and its Supplementary Information files.
