# Supplementary material for: Accumulation of protein aggregates induces autolytic programmed cell death in hybrid tobacco cells expressing hybrid lethality
Source: Sci Rep. 2019 Jul 15;9:10223. doi: 10.1038/s41598-019-46619-5 (PMC6629611; doi:10.1038/s41598-019-46619-5)

**Accumulation of protein aggregates induces autolytic programmed cell death in hybrid tobacco cells expressing hybrid lethality**

**Naoya Ueno^1^, Megumi Kashiwagi^1^, Motoki Kanekatsu^1^, Wataru Marubashi^2^, Tetsuya Yamada^1^**

^1^ Graduate School of Agricultural Science, Tokyo University of Agriculture and Technology, Tokyo, Japan

^2^ Faculty of Agricultural Science, Meiji University, Kanagawa, Japan

Address correspondence to: Tetsuya Yamada, e-mail: teyamada@cc.tuat.ac.jp, Phone/Fax: +81-42-367-5683

**Figure S1** Confirmation of proteasome inhibition by MG-132 in hybrid cells. Proteasome activity was measured in hybrid cells for cultures treated with MG-132 after 6 h at 28 °C. Asterisks indicate significant differences from DMSO control (***P* < 0.01, Student's *t*-test).

**Table S1** Summary of proteins identified from all experiment of proteome analysis of insoluble proteins.

**Table S2** Summary of proteins identified as insoluble proteins accumulated specifically in the incubation with E-64 in 28°C.

**Table S3** Summary of proteins identified as insoluble proteins accumulated specifically in the incubation with E-64 in 36°C.

**Table S4** GO of the identified proteins accumulated specifically in the incubation with E-64 in 28°C (i) and 36°C (ii).

**Table S5** Pathway of the identified proteins accumulated specifically in the incubation with E-64 in 28°C (i) and 36°C (ii).

**Supplementary Figure S1**


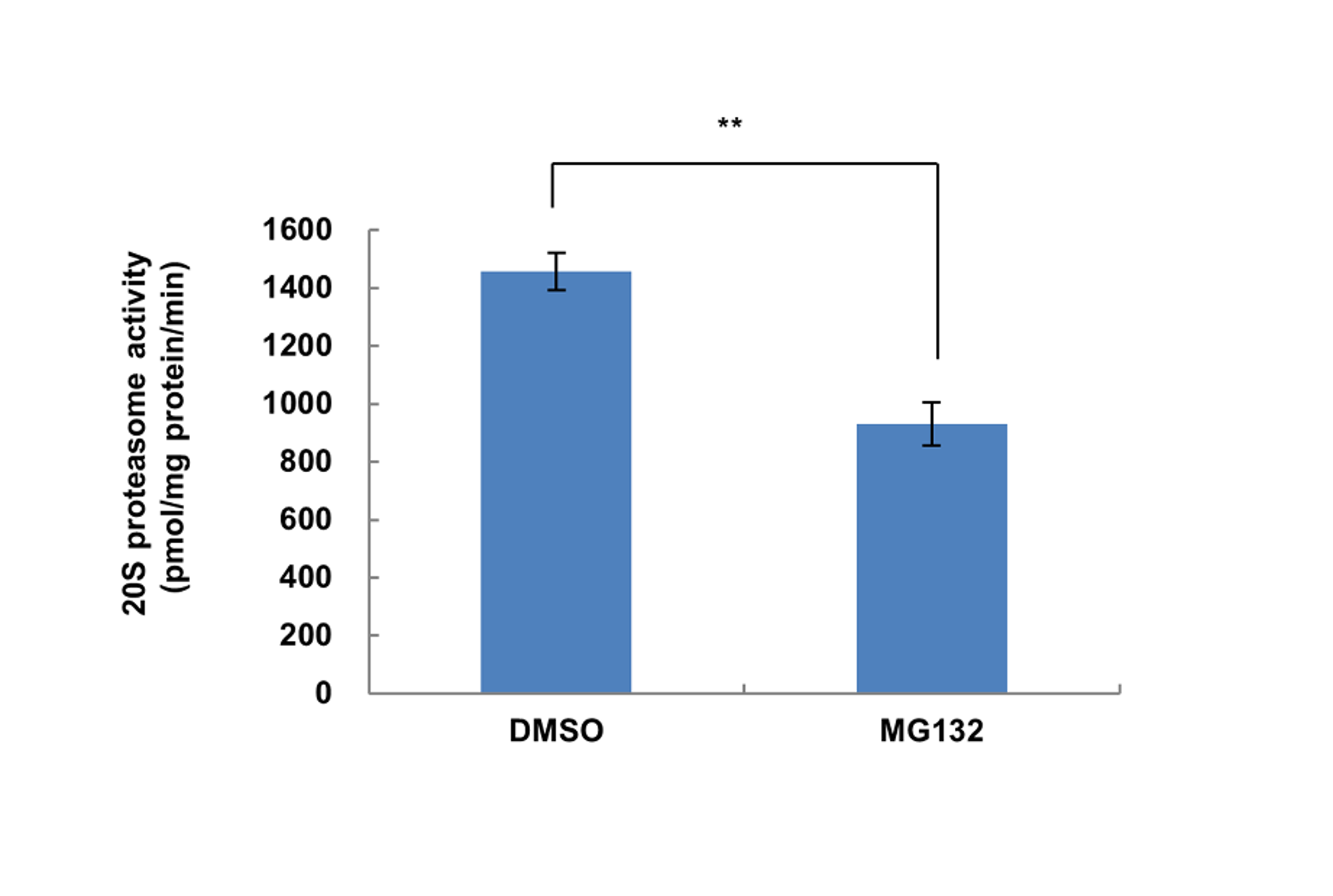

Supplement: Supplementary file 1 — Supporting Information [file 41598_2019_46619_MOESM1_ESM.docx]
